# Maintenance of the Neuroprotective Function of the Amino Group Blocked Fluorescence-Agmatine

**DOI:** 10.1007/s11064-021-03319-9

**Published:** 2021-04-29

**Authors:** Sumit Barua, A Young Sim, Jong Youl Kim, Injae Shin, Jong Eun Lee

**Affiliations:** 1 Department of Anatomy, Yonsei University College of Medicine, 50-1 Yonsei-Ro, Seodaemun-gu, Seoul, 03722 Korea; 2 BK21 Plus Project for Medical Sciences, Yonsei University College of Medicine, Seoul, 03722 Korea; 3 Brain Research Institute, Yonsei University College of Medicine, Seoul, 03722 Korea; 4 Department of Chemistry, Yonsei University, Seoul, 03722 Korea

**Keywords:** Agmatine, Guanidine, Neuroprotection, NMDA

## Abstract

Agmatine, an endogenous derivative of arginine, has been found to be effective in treating idiopathic pain, convulsion, stress-mediated behavior, and attenuate the withdrawal symptoms of drugs like morphine. In the early stages of ischemic brain injury in animals, exogenous agmatine treatment was found to be neuroprotective. Agmatine is also considered as a putative neurotransmitter and is still an experimental drug. Chemically, agmatine is called agmatine 1-(4-aminobutyl guanidine). Crystallographic study data show that positively-charged guanidine can bind to the protein containing Gly and Asp residues, and the amino group can interact with the complimentary sites of Glu and Ser. In this study, we blocked the amino end of the agmatine by conjugating it with FITC, but the guanidine end was unchanged. We compared the neuroprotective function of the agmatine and agmatine-FITC by treating them in neurons after excitotoxic stimulation. We found that even the amino end blocked neuronal viability in the excitotoxic condition, by NMDA treatment for 1 h, was increased by agmatine-FITC, which was similar to that of agmatine. We also found that the agmatine-FITC treatment reduced the expression of nitric oxide production in NMDA-treated cells. This study suggests that even if the amino end of agmatine is blocked, it can perform its neuroprotective function.

**Supplementary Information:**

The online version contains supplementary material available at 10.1007/s11064-021-03319-9.

## Introduction

Primary amine agmatine can be created naturally from α-amino l-arginine. The decarboxylation of l-arginine to produce agmatine occurs in the presence of the mammalian mitochondrial outer membrane enzyme arginine decarboxylase. Agmatine is catabolized into prototypical polyamine putrescine by agmatinase, a human ureohydrolase. Agmatine has been found in different organs of the body at lower concentrations, with enrichments in a few parts of the brain and spinal cord, packaged in the synaptic vesicles [1–3]. n the synaptic vesicles, agmatine is found to be co-localized with other classical neurotransmitters such as glutamate and vasopressin and can be released by calcium-dependent depolarization [4, 5]. With the above characteristics, agmatine is believed to be a neuromodulator that can act as a neurotransmitter, and localized in the oxytocin and vasopressin neurons [6, 7].

Agmatine was found to be neuroprotective in different excitotoxic and ischemic neurological diseases. Agmatine has been reported to reduce vascular permeability in the brain; this induces gastric protection in ischemic-injury rats [8]. The neuroprotective mechanism of agmatine is not yet clearly understood; however, it is believed that during the early hours of the ischemic/traumatic injury, activation of the unabated inducible nitric oxide synthase (iNOS) occurs, which increases nitric oxide (NO) production, whereas the level of arginine decarboxylase (ADC)/agmatine remains low [9–11]. In this condition, the sustained higher level of NO opens the N-methyl-d-aspartate (NMDA) channels through which the calcium influxes, causing neurotoxicity. After several hours of injury, the ADC/agmatine level increases and controls the iNOS and NMDA receptor functions [12, 13]. In addition to ischemic or traumatic injury, agmatine has been found to have a positive effect on neurological diseases such as Alzheimer’s disease, Parkinson’s disease, epilepsy, and other mental diseases [14–16]. Agmatine was also reported to be anti-apoptotic, an effect it achieves by attenuating the expression of caspase-3 and Bax, and elevating the expression of BCL2, Nrf2, and PI3K. [15, 17–20]. In a recent neurochemical profiling study, acute and sub-chronic oral treatment of agmatine was reported to be well tolerated and did not show any adverse effect in APPswe/PS1ΔE9 transgenic (Tg) mice, and also found to cross the blood brain barrier and accumulate in the brain [21].

Agmatine was first believed to be a clonidine-displacing substance that specifically binds to α2-adrenoceptors, I1- and I2-binding sites [22]. However, later studies suggested that agmatine can function through other neurological receptors, such as N-methyl-D-aspartate receptor, 2-amino-3-(5-methyl-3-oxo-1,2-oxazol-4-yl) propanoic acid receptor, kainate receptor, acetylcholine receptor, and serotonin receptor [23]. The positively-charged guanidine and amino end of the agmatine tend to bind with the Gly and Asp residues of the protein and the Glu and Ser residues of the complementary sites of the protein, respectively [24]. These two ends in agmatine have been suggested to play a vital role in neuroprotection, but there is no specific report yet. In this study, we intended to visualize the exogenous agmatine by attaching a fluorescein isothiocyanate (FITC) to its amino end and studied the role of agmatine in in vitro neuron culture. Our goal was to check, if the modified fluorescence-agmatine still conserve its neuroprotective function as the normal agmatine and how?

## Material and Methods

### Chemistry of Agmatine-FITC

#### FITC-Agmatine

To a stirred solution of FITC (50 mg, 0.12 mmol) and TEA (18 *μ*L, 0.12 mmol) in DMF (1 mL) was added agmatine (0.16 mg, 0.12 mmol) at room temperature. After stirring for 3 h at room temperature, the reaction was quenched by the addition of H_2_O. The organic solution was washed with water and brine, dried over anhydrous Na_2_SO_4_, filtered, and concentrated under reduced pressure. The residue was purified by flash column chromatography (CH_2_Cl_2_: MeOH = 10:1) to give a product in 60% yield: ^1^H nuclear magnetic resonance (NMR) (400 MHz, DMSO-*d6*)^δ^.82 (brs, 1 H), 8.24 (s, 1 H), 8.03 (brs, 1 H), 7.78 (d, 1 H, *J* = 9.3 Hz), 7.28 (brs, 2 H), 7.11 (d, 1 H, *J* = 9.3 Hz), 6.73 (d, 2 H, *J* = 9.3 Hz), 6.52–6.48 (m, 4 H), 3.51–3.42 (m, 2 H), 3.12–3.03 (m, 2 H), 1.61–1.45 (m, 4 H); ESI–MS calcd for C_26_H_25_N_5_O_5_S [M]^+^ 519.1 found 519.1.

### Primary Neuronal Cell Culture

Primary neuron culture was done as previously reported [25]. Before primary neuronal cell culture, the plates were coated with Poly-d-Lysine (Gibco) containing laminin overnight at room temperature, and the dishes were washed three times with autoclaved distilled water. Brains were extracted from ICR mice E14.5 (Koatech). The pregnant mice were euthanized using ether. Before incision, the abdomen was wiped with 70% ethanol to prevent possible contamination, after which the skin was cut and removed, and the abdominal wall was incised. Fetuses were decapitated with a pair of scissors, and the heads were placed in a petri dish containing hank’s balanced salt solution (HBSS). When the skull was opened, the olfactory bulbs, meninges, and hippocampus were removed systematically, and the cortex was isolated. The cells were pelleted by centrifugation at 1000×*g* for 3 min, and the supernatant was removed. The cells were mechanically dissociated using a Pasteur pipette (20 times). Approximately 1 × 10^6^ cells/mL were plated on PDL-laminin-coated 6-well plates in a neurobasal medium (Gibco) containing l-glutamine (Hyclone), penicillin–streptomycin (Hyclone), and B27 supplement (Gibco), and then cultured at 37 °C in 5% C02/95% air. After 7 days in vitro, the cells were treated with NMDA (50 μM), agmatine (100 μM), and agmatine-FITC (AgmF, 100 μM). To check the cellular uptake of the AgmF, cells were treated with AgmF and CellTracker™ Red CMTPX Dye (Thermo Fisher, cat: C34552). All animal experiments were conducted in accordance with the guidelines on the use and care of laboratory animals established by the Animal Care Committee Yonsei University.

### Immunocytochemistry

Neurons were washed three times with iced-PBS and permeabilized with 4% PFA. The cells were then incubated with primary antibodies at 4 °C overnight. The following primary antibodies were used: DCX (1:500), NOS2 (1:200). The primary antibody was then removed, and the cells were washed three times for 3 min with PBST (10% with Trionx-100). The cells were incubated with FITC-conjugated anti-IgG (1:500) and rhodamine-conjugated anti-IgG (1:500) at room temperature for 2 h. After washing three times for 3 min with PBS, the cells were counterstained with DAPI (1:500) for 10 min at room temperature. They were imaged using a Zeiss LSM 700 confocal microscope (Carl Zeiss, Thornwood, NY, USA). For each immunochemistry experiments 3–5 independent experiments were performed and 4–5 images from each experiment were analyzed while counting the cell number.

### Crystal Violet Staining

To check the viability of the adherent neurons after the NMDA and AgmF treatment we performed crystal violet staining of the neurons. After treating the cells with NMDA followed by Agmatine and AgmF we washed the cells with PBS for 2 times and stained the cells with crystal violet (0.5% in water/methanol 4/1) for 5 min and then rinsed with water and left to dry overnight [26]. The images were taken by using a light microscopy. Cell number was counted from 4–5 images obtained from 4 independent experiments.

### MTT Assay

Neurons (1 × 10^6^ cells/mL) were seeded in 24-well plates to monitor all experimental conditions. The cells were then treated with NMDA (50 μM) for 1 h followed by a PBS wash, agmatine, and AgmF for 6 h. Next, cells were rinsed twice with PBS, and the culture medium was replaced with neurobasal medium (Gibco). Then, 100 μL of 3-(4,5-dimethylthiazol-2-yl)-2,5-diphenyltetrazolium bromide (MTT, Sigma) solution (5 mg/mL in PBS) was added to each well. After 1 h of incubation, the medium was removed, and dimethyl sulfoxide was added to solubilize the purple formazan product of the MTT reaction. The supernatant from each well was analyzed using an enzyme-linked immunosorbent assay plate reader at a wavelength of 570 nm, with background subtraction at 650 nm. All experiments were repeated at least three times. Cell viability in the control medium without any treatment was considered 100%. Cell viability was reported as the value relative to the control group.

### Nitric Oxide Production in the Neurons

The neurons obtained from the ICR mice were cultured in a neurobasal medium (Gibco) containing 1% l-glutamine (Hyclone), 1% penicillin–streptomycin (Hyclone), and B27 supplement (Gibco). The cells were plated at a density of 1 × 106, pre-incubated for 24 h at 37 °C, and maintained in a humidified atmosphere containing 5% CO_2_. The mammalian cells were treated with NMDA (50 μM) for 1 h and then washed 3 times with PBS to remove the NMDA and treated with agmatine and AgmF for 6 h in culture media. Accumulated nitrite (NO_2_−), as an index of nitric oxide (NO), in the media was determined using a colorimetric assay based on the Griess reaction. After 6 h cells were washed with PBS and fixed with 4% PFA and stored at 4 °C until NOS2 staining.

### Statistical Analysis

The data are quantified as the mean ± SEM from at least three different experiments performed from separate cell preparations, and at least quadruplicate determinations were performed in each experiment. Statistical tests to determine differences between groups others were analyzed with analysis of variance followed by Tukey’s post hoc test. SigmaPlot 12.0 (Systat Software Inc., San Jose, California, USA) for Windows was used. p values less than 0.05 were considered significant.

## Results

### Chemistry of Agmatine-FITC and Neuronal Uptake

#### General

**Fig. 1:**
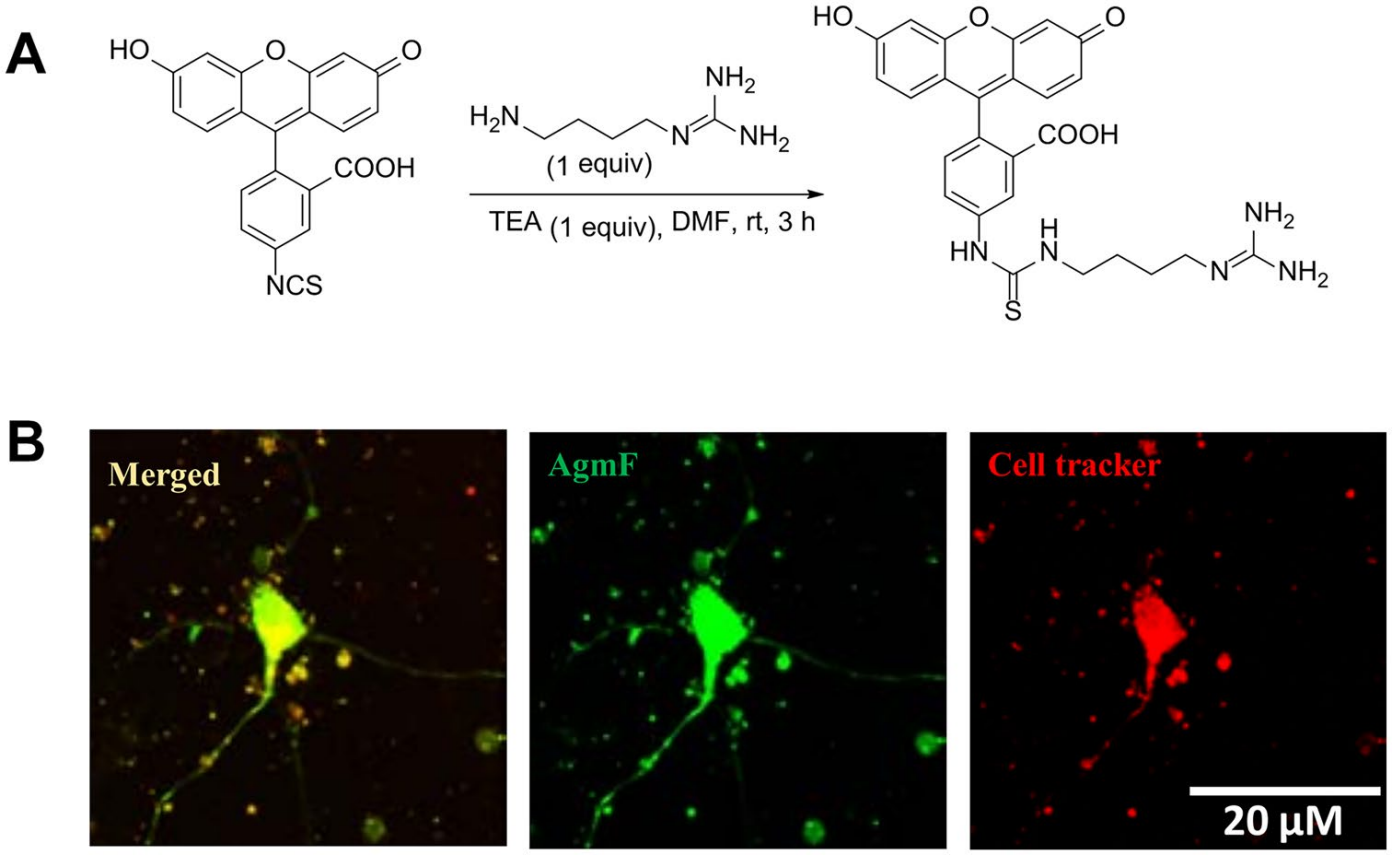
Preparation of Agmatine-FITC. **a** chemical reaction of FITC bind to the free amino end of Agm. **b** Cellular uptake of the AgmF was confirmed in primary cortical neurons by stained with cell tracker. Scale bar: 20 μm

Analytical thin-layer chromatography was conducted on silica gel in 60 F254 glass plates. Compound spots were visualized by ultraviolet light (254 nm) and/or by staining with 10 wt% phosphomolybdic acid in ethanol. Flash column chromatography was performed using silica gel 60 (230–400 mesh). NMR spectra were recorded on a Bruker DRX-400 instrument. Mass spectra were obtained using a Waters 3100 LC/MS System. Chemical reaction showing the making of agmatine-FITC (Fig. 1a). To confirm the cellular uptake of the AgmF, we treated the cultured neurons with AgmF (green) for 6 h, and then CellTracker™ Red CMTPX Dye (red) and confocal images were taken (Fig. 1b). Green AgmF was found in both the cell body and cell neurite.

### AgmF can Protect the Neuronal Cells from NMDA-Induced Neurotoxicity

**Fig. 2:**
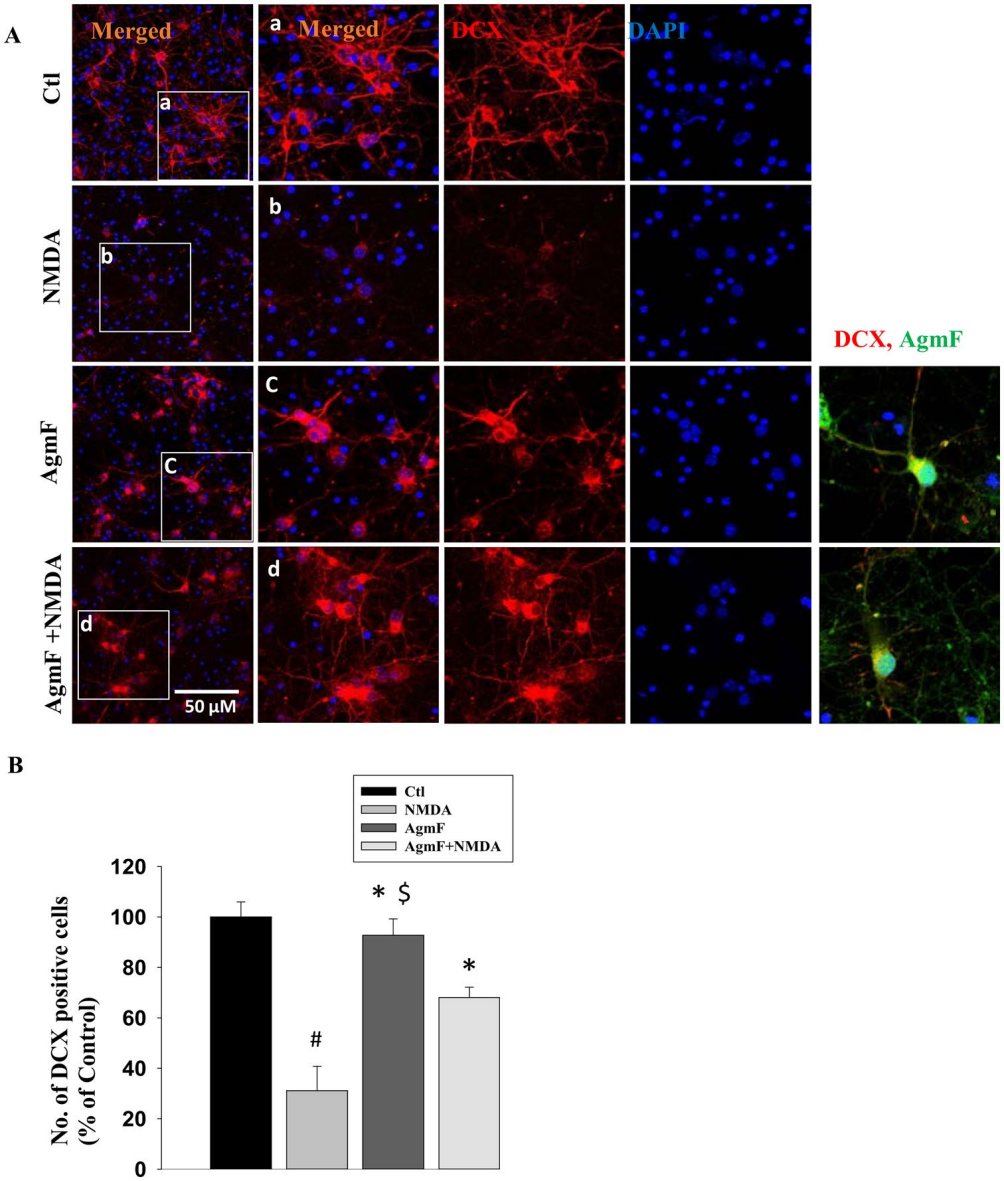
Cellular damage protection by Agmatine-FITC (AgmF). DI7 cortical cells were treated with NMDA (50 µM for 1 h) followed by and AgmF (100 µM) for 6 h. **a** After 6 h, cortical cells showed healthy cell bodies with long neurites. **A** The NMDA treatment showed that the number of cells with neurites gradually reduced with time. The **a**–**d** are the 4 × enlarged images of the corresponding white square boxed images. **B** The treatment of AgmF with or without NMDA groups showed that the number of cells with neurites and cell bodies was significantly higher than that in the NMDA-treated cells. Scale bar: 50 μm. *P < 0.001 versus the NMDA, ^#^p < 0.001 versus the cortical and ^$^p < 0.01 versus NMDA + AgmF.

NMDA receptors are found in the neuron cell body, and synapses are responsible for important physiological and pathological conditions of neurons. Depending on the NMDA treatment concentration, NMDA receptors can regulate the intercellular response bi-directionally. Lower and higher NMDA concentrations in NMDA treatment can activate the survival and pro-death signaling in neurons [27]. To check the role of AmgF in cortical neurons, we treated the neurons with a higher concentration of NMDA (50 µM) for 1 h, followed by AgmF (100 µM) for 6 h, and fixed cells were immunostained with the neuron marker DCX. We found that AgmF itself does not have any effect on neurons. Treatment with NMDA reduced the number of DCX-positive cells to 31% as expected when compared with the control (100%). However, NMDA-treated cells, followed by AgmF, were approximately 37% higher than the NMDA-only treated group (Fig. 2a, b). The above results suggest that AgmF does not have any harmful effect on neurons and is neuroprotective in high NMDA treatment.

### Comparison of the Neuroprotective Effects Between Agmatine and AgmF

**Fig. 3:**
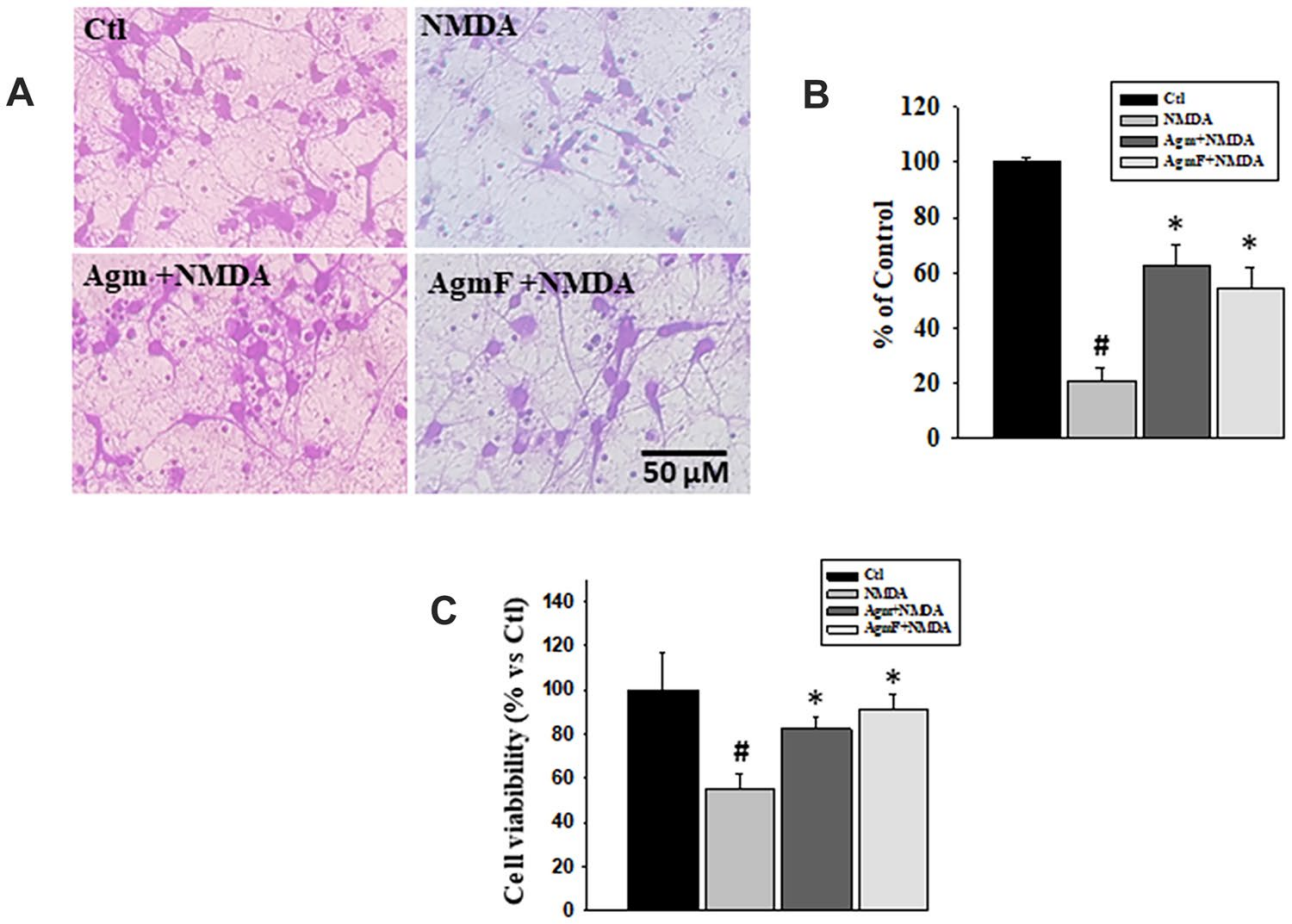
Neuroprotection by Agmatine (Agm) and AgmF. DIV7 cortical cells were treated with NMDA (50 µM) followed by Agm and AgmF (100 µM) for 6 h and then fixed and stained with crystal violet. **a** after 6 h, control cells showed healthy cell bodies with long neurites; however, NMDA showed a significantly lower number of cells with neurites, whereas the Agm and AgmF-treated groups showed a healthy cell body with longer neurites compared to NMDA treatment. **b** Crystal violet-stained cells were counted. **c** MTT assay showed the same result as found in crystal violet staining. Scale bar: 50 μm. *P < 0.001 compared to the control.

The physiological function of endogenous agmatine is largely unknown. However, several studies have reported that exogenous agmatine treatment can reduce neuronal injury in both in vitro and in vivo disease models. With this view, we investigated the changes in neuroprotection by agmatine when the amino end is blocked, and only the guanidine end is active. In this investigation, NMDA-treated cells were treated with agmatine and AgmF for 6 h. Crystal violet staining of the fixed cells showed that agmatine (63% of the control) had a greater number of cells (about 41% more) than the NMDA (21% of control) (Fig. 3a, b). The AmgF (about 54% of the control) treatment also has a significantly higher (about 33% more) number of cells than the NMDA-treated cells. However, the difference in the cell number between agmatine and AgmF was not significant, at approximately 8%. Again, in the MTT assay, the NMDA treatment reduced the cell viability to 54%, whereas NMDA treatment followed by agmatine and AgmF maintained the cell viability at 82% and 91%, respectively, compared to the control; this was significantly higher than that of the NMDA-only treatment (Fig. 3c). The above results suggest that there was no significant difference in the neuroprotective functions of agmatine and AgmF.

### AgmF Reduced no Production by Attenuating NOS2 Expression

**Fig. 4:**
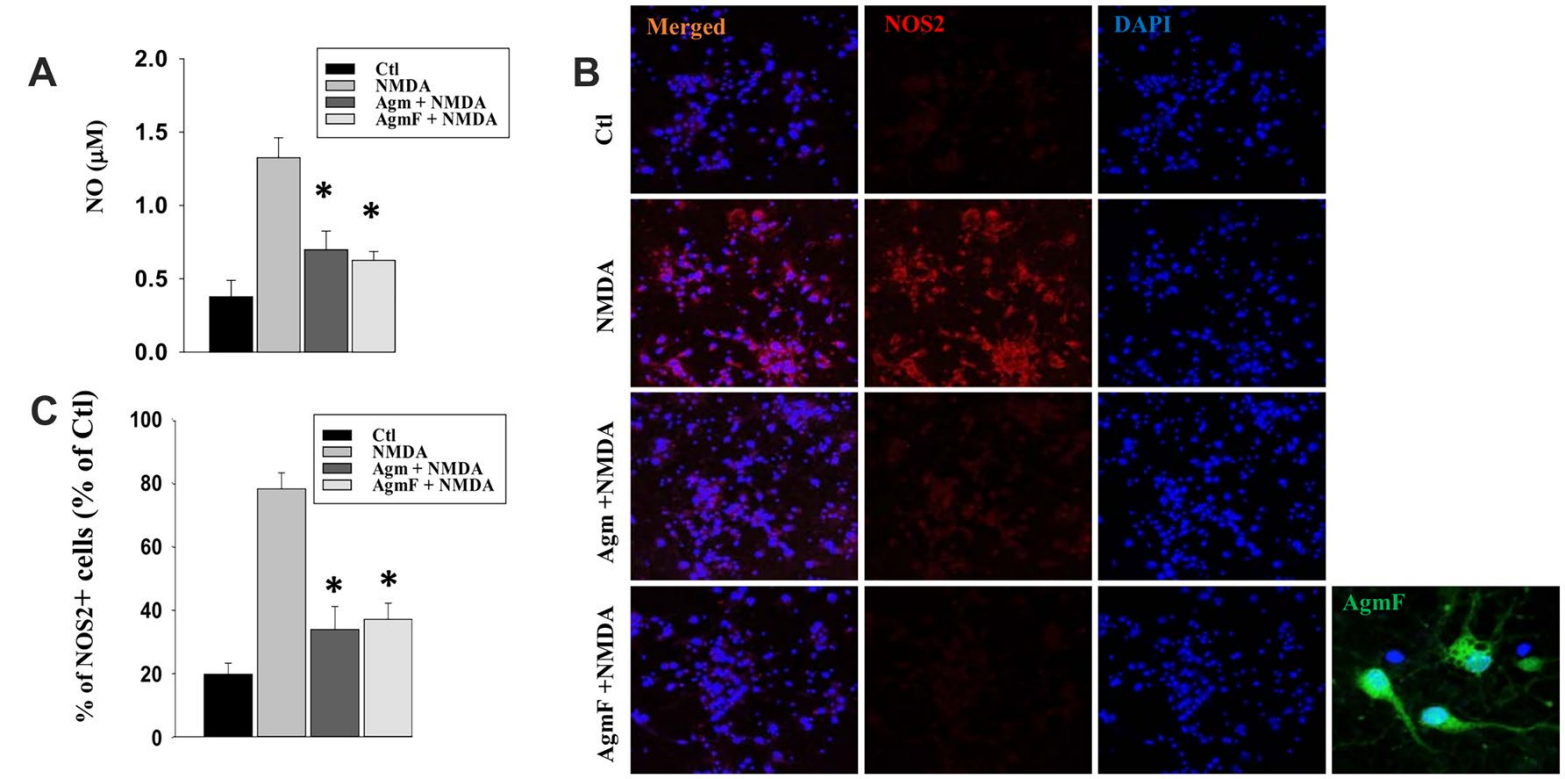
AgmF reduces nitric oxide (NO) production via inhibition of NOS2 expression. **a** In the NMDA DIV7 cortical cells, NO production was reduced after 6 h of treatment with Agm and AgmF. **b** NMDA-induced NOS2 expression was also reduced by Agm and AgmF treatment. **c** the NOS2^+^ neurons are counted. Scale bar: 50 μm. *P < 0.001 compared to the control.

One of the mechanisms of neuroprotection by agmatine is the reduction of NO production. To investigate the involvement of this mechanism in AgmF-mediated neuroprotection, we performed the NO assay after treating the cells with NMDA, agmatine, and AgmF. NMDA treatment increased NO production (1.3 μM) by about fivefold compared to the control (0.3 μM). However, both agmatine and AgmF treatment significantly reduced NO production (0.7 and 0.6 μM) when compared with NMDA (Fig. 4a). Immune staining of the cells with NOS2 showed that NOS2 expression was increased by NMDA treatment; however, both agmatine and AgmF treatment followed by NMDA treatment significantly reduced NOS2 expression (Fig. 4b). The NOS^+^ neurons are counted and found that NMDA has increased the NOS2 expressing cells about 60% more than the control cells. However, the agmatine and the AgmF treatment have reduced the number of the NOS^+^ about 50% of the NMDA treated cells (Fig. 4c). The above results show that AgmF exerts neuroprotective effects by reducing NO production, which is achieved by inhibiting NOS2 expression.

## Discussion

In our study, we demonstrated the procedure of making a fluorescence agmatine by biding a FITC molecule at the amino end of the agmatine and the guanidine end remains active. After producing the fluorescence-agmatine we also evaluated that it protects against NMDA-mediated injury in neuronal cultures. Furthermore, even with the above modification of the agmatine structure, the quality of the neuroprotective function remains unchanged.

Agmatine-expressing cells have been found in all regions of the brain, such as the hypothalamus, frontal cortex, striatum, medulla, hippocampus, and locus coeruleus (LC), with measurable ADC activity [28]. However, the highest number of agmatine-expressing cells was found in the hypothalamus, which also had the highest ADC activity [28, 29]. There are diverse mechanisms of neuroprotection in different neurodegenerative diseases by agmatine that have been reported by different research groups [16, 30, 31] and also by us. Agmatine belongs to the class of organic compounds known as guanidines. Structurally, agmatine has two ends that consist of a guanidine moiety and an amino group [32]. In this study, we blocked the amino group by binding a FITC molecule and confirmed the binding of agmatine to FITC by NMR. The binding of FITC to the amino group did not change the neuronal uptake of agmatine. Although the actual function of endogenous agmatine has not yet been discovered, it is well known that exogenous agmatine can act as a neurotransmitter and protect neurons in different diseases such as AD, PD, ischemic stroke, traumatic brain injury, spinal cord injury, etc. [4, 14, 33–35]. In pathological conditions, increased extracellular glutamate over-activates the NMDARs, resulting in elevated Ca^2+^ and Na^+^ influx into the cell, which triggers NMDAR-mediated neuronal excitotoxicity and causes neuronal cell death [36–38]. In our study, we found that NMDA treatment reduced the number of DCX-positive cells, but there was no change in the AgmF-treated cells. Moreover, AgmF inhibited the cytotoxic effect of NMDA. Therefore, even if the free amino end was blocked, agmatine would not be harmful to the neurons. Given that agmatine and AgmF might differ with respect to neuroprotection, we studied neuronal viability by agmatine and AgmF following NMDA treatment. The neuronal viability by AgmF was found to be similar to that of agmatine.

NMDAR over-activation by exogenous NMDA treatment produces NO by activating NOS, which is a major mediator of neuronal death [39–41]. As a result, controlling neuronal excitotoxicity and the inhibition of NO through the modulation of NMDAR functions resulted in the prior choice of treatment for ion channel-related brain disorders. Primary amine agmatine has been reported to be a neuroprotective agent by modulating the NMDAR, NO pathway, and oxidative stress in various neurodegenerative diseases [10, 39, 40]. In hypoxic microglia, agmatine attenuates NO production by inhibiting the expression of iNOS [41]. The endogenous neurotransmitter agmatine is a selective blocker of NMDAR [23, 40]. In our study, we found that both agmatine and AgmF treatment reduced the production of NO using NMDA by half, and there was no significant difference in NO production between them. The reduction of NO production was found to be mediated by the reduction of the activity of the enzyme NOS2. Several studies previously suggested that the reduction of NO production and NOS2 inhibition by agmatine was mediated through selective blockage of the NMDA subclass of glutamate receptors [42]. In our study, NO production and NOS2 inhibition by agmatine and AgmF were found to be similar, suggesting that there were no changes in the selective blockage of the NMDA subclass of glutamate receptors after blocking the free amino end of agmatine.

In conclusion, we suggest that the guanidine end of agmatine regulates its neuroprotective function. As of today, there is no agmatine conjugated with a fluorescent compound. Our modified FITC AgmF can be used in studies in which fluorescence agmatine is needed.

## Supplementary Information

Below is the link to the electronic supplementary material.

Supplementary file1 Supplementary Figure 1 MAP2 staining of the neuron treated with AgmF with or without NMDA to check the role of agmatine on neuron. NMDA was treated as a positive control. (TIF 2109 kb)

## Data Availability

The datasets generated during and analysed during the current study are available from the corresponding author on reasonable request.

## References

[1] Molderings GJ, Heinen A, Menzel S, Lubbecke F, Homann J, Gothert M (2003). Gastrointestinal uptake of agmatine: distribution in tissues and organs and pathophysiologic relevance. Ann N Y Acad Sci.

[2] Raasch W, Regunathan S, Li G, Reis DJ (1995). Agmatine, the bacterial amine, is widely distributed in mammalian tissues. Life Sci.

[3] Raasch W, Schafer U, Chun J, Dominiak P (2001). Biological significance of agmatine, an endogenous ligand at imidazoline binding sites. Br J Pharmacol.

[4] Park YM, Lee WT, Bokara KK, Seo SK, Park SH, Kim JH, Yenari MA, Park KA, Lee JE (2013). The multifaceted effects of agmatine on functional recovery after spinal cord injury through Modulations of BMP-2/4/7 expressions in neurons and glial cells. PLoS ONE.

[5] Reis DJ, Regunathan S (2000). Is agmatine a novel neurotransmitter in brain?. Trends Pharmacol Sci.

[6] Goracke-Postle CJ, Overland AC, Stone LS, Fairbanks CA (2007). Agmatine transport into spinal nerve terminals is modulated by polyamine analogs. J Neurochem.

[7] Gorbatyuk OS, Milner TA, Wang G, Regunathan S, Reis DJ (2001). Localization of agmatine in vasopressin and oxytocin neurons of the rat hypothalamic paraventricular and supraoptic nuclei. Exp Neurol.

[8] Al Masri AA, El Eter E (2012). Agmatine induces gastric protection against ischemic injury by reducing vascular permeability in rats. World J Gastroenterol.

[9] Feng Y, Piletz JE, Leblanc MH (2002). Agmatine suppresses nitric oxide production and attenuates hypoxic-ischemic brain injury in neonatal rats. Pediatr Res.

[10] Mun CH, Lee WT, Park KA, Lee JE (2010). Regulation of endothelial nitric oxide synthase by agmatine after transient global cerebral ischemia in rat brain. Anat Cell Biol.

[11] Zhao X, Ross ME, Iadecola C (2003). L-Arginine increases ischemic injury in wild-type mice but not in iNOS-deficient mice. Brain Res.

[12] Kim JH, Kim JY, Jung JY, Lee YW, Lee WT, Huh SK, Lee JE (2017). Endogenous agmatine induced by ischemic preconditioning regulates ischemic tolerance following cerebral ischemia. Exp Neurobiol.

[13] Satriano J, Schwartz D, Ishizuka S, Lortie MJ, Thomson SC, Gabbai F, Kelly CJ, Blantz RC (2001). Suppression of inducible nitric oxide generation by agmatine aldehyde: beneficial effects in sepsis. J Cell Physiol.

[14] El-Sayed EK, Ahmed A, Morsy EE, Nofal S (2019). Neuroprotective effect of agmatine (decarboxylated l-arginine) against oxidative stress and neuroinflammation in rotenone model of Parkinson's disease. Hum Exp Toxicol.

[15] Song J, Hur BE, Bokara KK, Yang W, Cho HJ, Park KA, Lee WT, Lee KM, Lee JE (2014). Agmatine improves cognitive dysfunction and prevents cell death in a streptozotocin-induced Alzheimer rat model. Yonsei Med J.

[16] Xu W, Gao L, Li T, Shao A, Zhang J (2018). Neuroprotective role of agmatine in neurological diseases. Curr Neuropharmacol.

[17] Arndt MA, Battaglia V, Parisi E, Lortie MJ, Isome M, Baskerville C, Pizzo DP, Ientile R, Colombatto S, Toninello A, Satriano J (2009). The arginine metabolite agmatine protects mitochondrial function and confers resistance to cellular apoptosis. Am J Physiol Cell Physiol.

[18] Chai J, Luo L, Hou F, Fan X, Yu J, Ma W, Tang W, Yang X, Zhu J, Kang W, Yan J, Liang H (2016). Agmatine reduces lipopolysaccharide-mediated oxidant response via activating PI3K/Akt pathway and up-regulating Nrf2 and HO-1 expression in macrophages. PLoS ONE.

[19] Freitas AE, Egea J, Buendia I, Navarro E, Rada P, Cuadrado A, Rodrigues AL, Lopez MG (2015). Agmatine induces Nrf2 and protects against corticosterone effects in hippocampal neuronal cell line. Mol Neurobiol.

[20] Wang CC, Chio CC, Chang CH, Kuo JR, Chang CP (2010). Beneficial effect of agmatine on brain apoptosis, astrogliosis, and edema after rat transient cerebral ischemia. BMC Pharmacol.

[21] Bergin DH, Jing Y, Williams G, Mockett BG, Zhang H, Abraham WC, Liu P (2019). Safety and neurochemical profiles of acute and sub-chronic oral treatment with agmatine sulfate. Sci Rep.

[22] Li G, Regunathan S, Barrow CJ, Eshraghi J, Cooper R, Reis DJ (1994). Agmatine: an endogenous clonidine-displacing substance in the brain. Science.

[23] Barua S, Kim JY, Kim JY, Kim JH, Lee JE (2019). Therapeutic effect of agmatine on neurological disease: focus on ion channels and receptors. Neurochem Res.

[24] Osawa T, Kimura S, Terasaka N, Inanaga H, Suzuki T, Numata T (2011). Structural basis of tRNA agmatinylation essential for AUA codon decoding. Nat Struct Mol Biol.

[25] Song J, Lee JH, Lee SH, Park KA, Lee WT, Lee JE (2013). TRPV1 activation in primary cortical neurons induces calcium-dependent programmed cell death. Exp Neurobiol.

[26] Andreoni G, Angeretti N, Lucca E, Forloni G (1997). Densitometric quantification of neuronal viability by computerized image analysis. Exp Neurol.

[27] Zhou X, Hollern D, Liao J, Andrechek E, Wang H (2013). NMDA receptor-mediated excitotoxicity depends on the coactivation of synaptic and extrasynaptic receptors. Cell Death Dis.

[28] Iyo AH, Zhu MY, Ordway GA, Regunathan S (2006). Expression of arginine decarboxylase in brain regions and neuronal cells. J Neurochem.

[29] Mella C, Martinez F, de Los Angeles Garcia M, Nualart F, Castro V, Bustos P, Carvajal N, Uribe E (2010). Expression and localization of an agmatinase-like protein in the rat brain. Histochem Cell Biol.

[30] Kim JY, Lee YW, Kim JH, Lee WT, Park KA, Lee JE (2015). Agmatine attenuates brain edema and apoptotic cell death after traumatic brain injury. J Korean Med Sci.

[31] Kotil K, Kuscuoglu U, Kirali M, Uzun H, Akcetin M, Bilge T (2006). Investigation of the dose-dependent neuroprotective effects of agmatine in experimental spinal cord injury: a prospective randomized and placebo-control trial. J Neurosurg Spine.

[32] Huang MJ, Regunathan S, Botta M, Lee K, McClendon E, Yi GB, Pedersen ML, Berkowitz DB, Wang G, Travagli M, Piletz JE (2003). Structure-activity analysis of guanidine group in agmatine for brain agmatinase. Ann N Y Acad Sci.

[33] Kim DJ, Kim DI, Lee SK, Suh SH, Lee YJ, Kim J, Chung TS, Lee JE (2006). Protective effect of agmatine on a reperfusion model after transient cerebral ischemia: temporal evolution on perfusion MR imaging and histopathologic findings. AJNR Am J Neuroradiol.

[34] Kim JH, Yenari MA, Giffard RG, Cho SW, Park KA, Lee JE (2004). Agmatine reduces infarct area in a mouse model of transient focal cerebral ischemia and protects cultured neurons from ischemia-like injury. Exp Neurol.

[35] Zhu MY, Wang WP, Cai ZW, Regunathan S, Ordway G (2008). Exogenous agmatine has neuroprotective effects against restraint-induced structural changes in the rat brain. Eur J Neurosci.

[36] Brittain MK, Brustovetsky T, Brittain JM, Khanna R, Cummins TR, Brustovetsky N (2012). Ifenprodil, a NR2B-selective antagonist of NMDA receptor, inhibits reverse Na+/Ca2+ exchanger in neurons. Neuropharmacology.

[37] Dong XX, Wang Y, Qin ZH (2009). Molecular mechanisms of excitotoxicity and their relevance to pathogenesis of neurodegenerative diseases. Acta Pharmacol Sin.

[38] Gupta K, Hardingham GE, Chandran S (2013). NMDA receptor-dependent glutamate excitotoxicity in human embryonic stem cell-derived neurons. Neurosci Lett.

[39] Ruiz-Durantez E, Ruiz-Ortega JA, Pineda J, Ugedo L (2002). Effect of agmatine on locus coeruleus neuron activity: possible involvement of nitric oxide. Br J Pharmacol.

[40] Wang WP, Iyo AH, Miguel-Hidalgo J, Regunathan S, Zhu MY (2006). Agmatine protects against cell damage induced by NMDA and glutamate in cultured hippocampal neurons. Brain Res.

[41] Ahn SK, Hong S, Park YM, Lee WT, Park KA, Lee JE (2011). Effects of agmatine on hypoxic microglia and activity of nitric oxide synthase. Brain Res.

[42] Yang XC, Reis DJ (1999). Agmatine selectively blocks the N-methyl-D-aspartate subclass of glutamate receptor channels in rat hippocampal neurons. J Pharmacol Exp Ther.

