# Supplementary figures and images for: Maintenance of the Neuroprotective Function of the Amino Group Blocked Fluorescence-Agmatine
Source: Neurochem Res. 2021 Apr 29;46(8):1933–40. doi: 10.1007/s11064-021-03319-9 (PMC8254702; doi:10.1007/s11064-021-03319-9)

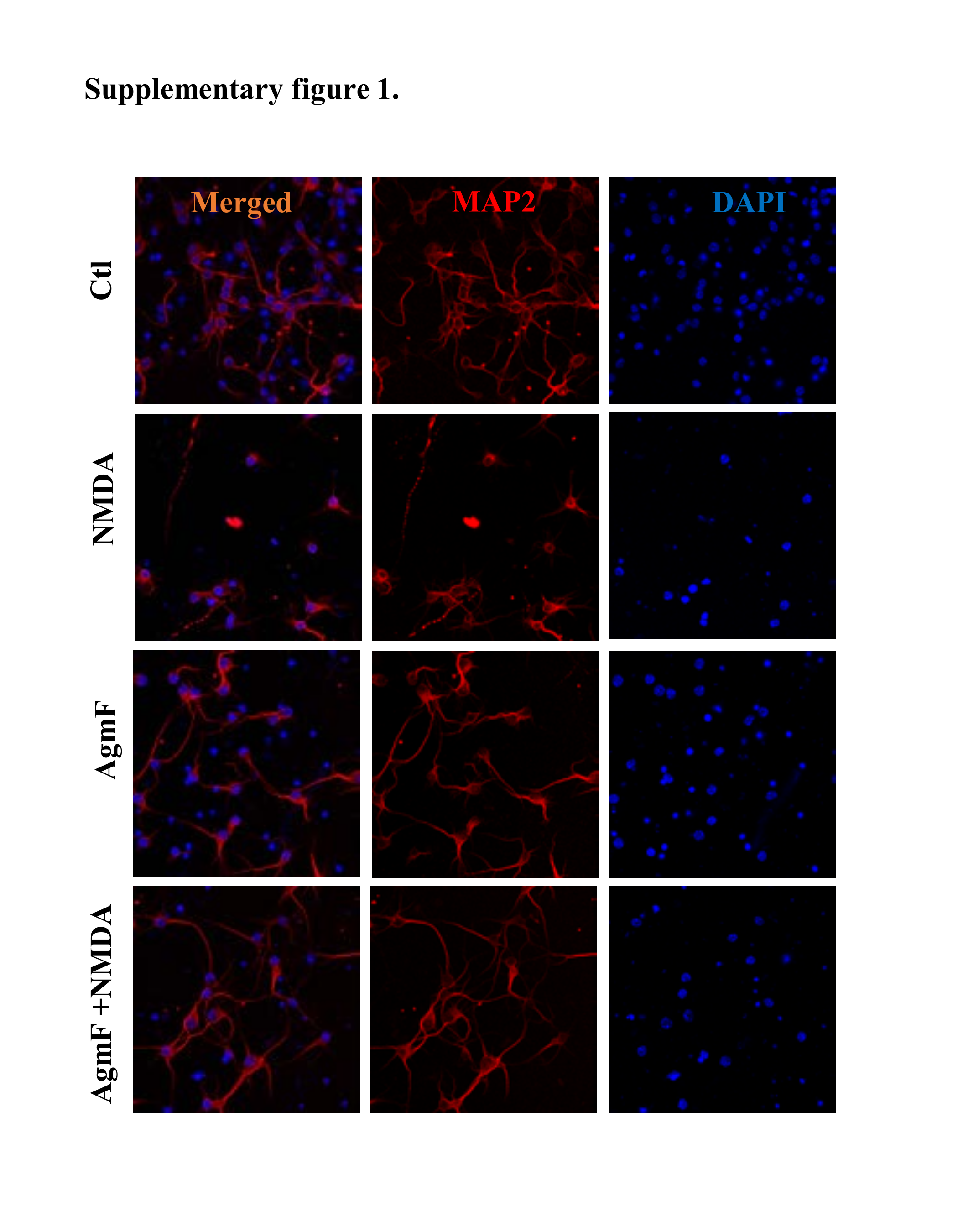

Supplement: Supplementary file 1 — Supplementary file1 Supplementary Figure 1 MAP2 staining of the neuron treated with AgmF with or without NMDA to check the role of agmatine on neuron. NMDA was treated as a positive control. (TIF 2109 kb) [file 11064_2021_3319_MOESM1_ESM.tif]
